# 

**Published:** 1880-12

**Authors:** 


### CONTENTS:

ART. I.—Filling Teeth—Some Special Points to be Observed. By E. Noyes, D. D. S. .......... 337

ART. II.—Carbolic Acid and Creasote—Their Chemistry and Therapeutical Application to the Practice of Dentistry. By Truman W. Brophy, M. D., D. D. S. .......... 348

ART. III.—Mechanical Dentistry. By E. M. Flagg, D. D. S. .......... 357

ART. IV.—The Preservation of all the Natural Teeth. By John T. Codman, D. D. S. .......... 363

ART. V.—Physical Examination of the Mouth and Throat—The Physical Signs Derivable from the Breath, Lips, Teeth and Mouth. By G. V. Poore, M. D., F. R. C. P. .......... 367

ART. VI.—Neuralgia of the Fifth Pair Cured by Aconite. By M. Landesberg, M. D. .......... 377

### EDITORIAL, ETC.

Pulp Capping .......... 379

The Celluloid Infringement .......... 379

The Fraudulent Diploma Man .......... 380

### BIBLIOGRAPHICAL.

The American Agriculturist .......... 381

The Scientific American .......... 381

A Practical Treatise on Mechanical Dentistry, (Richardson.) .......... 382

Atlas of Human Anatomy, (Wilde.) .......... 382

The Sanitarian .......... 383

### MONTHLY SUMMARY.

Toxic Effects of Carbolic Acid .......... 383

Fatal Carelessness .......... 384

Carbolic Acid Poisoning .......... 384

The Goodyear Patent .......... 384

